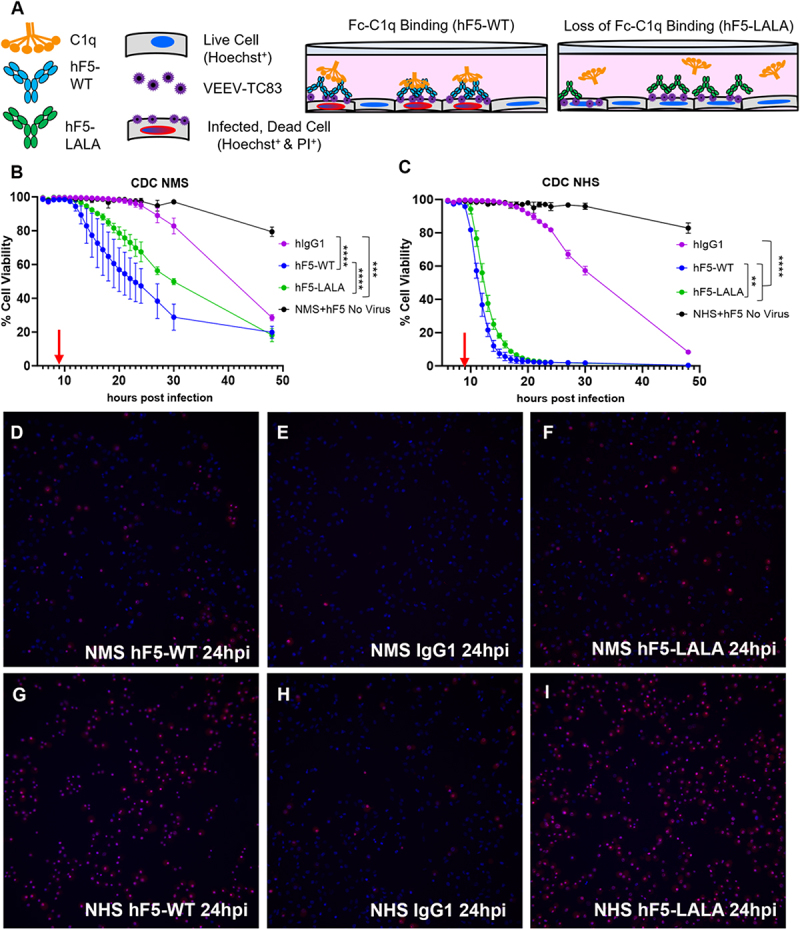# Correction

**DOI:** 10.1080/19420862.2024.2312050

**Published:** 2024-02-08

**Article title**: Therapeutic efficacy of a potent anti-Venezuelan equine encephalitis virus antibody is contingent on Fc effector function

**Authors**: Jennifer L. Schwedler, Maxwell A. Stefan, Christine E. Thatcher, Peter R. McIlroy, Anupama Sinha, Ashlee M. Phillips, Christopher A. Sumner, Colleen M. Courtney, Christina Y. Kim, Dina R. Weilhammer, and Brooke Harmon

**Journal**: *mAbs*

**DOI**: https://doi.org/10.1080/19420862.2023.2297451

The correct version of Figure 3 is as below.

**Figure:**